# Effect of suppressive acyclovir administered to HSV-2 positive mothers from week 28 to 36 weeks of pregnancy on adverse obstetric outcomes: a double-blind randomised placebo-controlled trial

**DOI:** 10.1186/s12978-017-0292-7

**Published:** 2017-03-03

**Authors:** Sarah Nakubulwa, Dan K. Kaye, Freddie Bwanga, Nazarius Mbona Tumwesigye, Edith Nakku-Joloba, Florence Mirembe

**Affiliations:** 1 Department of Obstetrics and Gynaecology, School of Medicine, Makerere University College of Health Sciences, P. O. Box 7072, Kampala, Uganda; 2 Department of Microbiology, Makerere University College of Health Sciences, P.O.Box 7072, Kampala, Uganda; 3 School of Public Health, Makerere University College of Health Sciences, P.O.Box 7072, Kampala, Uganda

**Keywords:** Acyclovir, Herpes simplex type 2, Genital herpes, Adverse obstetric outcomes, Pregnancy, Randomised controlled trial, Uganda

## Abstract

**Background:**

Acyclovir (ACV) given to HSV-2 positive women after 36 weeks reduces adverse outcomes but its benefit at lower gestation was undocumented. We determined the effect of oral acyclovir administered from 28 to 36 weeks on premature rupture of membranes (PROM) primarily and preterm delivery risk.

**Methods:**

This was a randomized, double-blind placebo-controlled trial among 200 HSV-2 positive pregnant women at 28 weeks of gestation at Mulago Hospital, Uganda. Participants were assigned randomly (1:1) to take either acyclovir 400 mg orally twice daily (intervention) or placebo (control) from 28 to 36 weeks. Both arms received acyclovir after 36 weeks until delivery. Development of Pre-PROM by 36 weeks and preterm delivery were outcomes.

**Results:**

One hundred women were randomised to acyclovir and 100 to placebo arms between January 2014 and February 2015. There was tendency towards reduction of incidence of PROM at 36 weeks but this was not statistically significant (4.0% versus 10.0%; RR 0.35; 95% 0.11–1.10) in the acyclovir and placebo arms respectively. However, there was a significant reduction in the incidence of preterm delivery (11.1% versus 23.5%; RR 0.41; 95% 0.20–0.85) in the acyclovir and placebo arms respectively.

**Conclusions:**

Oral acyclovir given to HSV-2 positive pregnant women from 28 to 36 weeks reduced incidence of preterm delivery but did not significantly reduce incidence of pre-PROM.

**Trial registration:**

www.pactr.org, PACTR201311000558197.

## Plain English summary

Acyclovir (a drug used to reduce genital herpes infection) when given to pregnant women with genital herpes from the 36 week causes reduction of undesirable outcomes to the mother or baby, although its effect when given earlier in pregnancy was undocumented. We aimed to determine the proportion of pregnant women with genital herpes who develop rupture of membranes before labour onset and the women who deliver prematurely when acyclovir is given to them from the 28 week to the 36 week.

Two hundred pregnant women with genital herpes at 28 weeks at Mulago Hospital, Uganda were allocated randomly in equal groups. One hundred women were given acyclovir 400 mg and 100 women were given a placebo tablet similar to acyclovir but without active ingredient for swallowing twice a day for 8 weeks. We monitored women for rupture of membranes before labour onset and for premature delivery.

Four of the 100 women on acyclovir compared to 10 of 100 women on the placebo tablet had rupture of membranes before labour onset although this was not substantial. Eleven of the 100 women on acyclovir compared to 24 of 100 women who on the placebo tablet had premature delivery which was substantial.

In conclusion, when acyclovir is given to pregnant women who have genital herpes from the 28 week to the 36 week of pregnancy, fewer women deliver prematurely although there is no substantial effect on the number of women developing rupture of membranes before labour onset.

## Background

Pregnant women who are *Herpes simplex* virus type 2 (HSV-2) seropositive are at risk of inflammation of both the genital tract and the trophoblastic tissue causing undesirable obstetric outcomes [1, 2]. Experimental studies have demonstrated that inflammatory mediators including cytokines are released when HSV-2 and other herpetic viruses invaded the cervical cells [3]. These inflammatory mediators among HSV-2 positive pregnancy women are known to cause premature rupture of membranes (PROM) [4–6]. Premature rupture of membranes is defined as rupture of membranes preceding commencement of regular uterine contractions signifying labour onset [4]. Rupture of membranes occurring at 37 weeks or more is term PROM and that before 37 weeks of gestation is referred to as pre- PROM [6, 7].

Globally, PROM complicates 3–10% of pregnancies [7, 8]. In a Nigerian Referral and Teaching Hospital in Enugu, 4.3% of pregnancies were complicated by PROM [9]. In Mulago Hospital, PROM complicates about 12% of women admitted in the labour ward in Mulago Hospital [10].

PROM is an undesired obstetric outcome which is often complicated by other adverse outcomes such as puerperal sepsis, fetal infection, fetal death and low Apgar score. Furthermore, pre-PROM is complicated by preterm delivery and low birth weight [6, 11]. PROM contributes to over a third of premature deliveries [7]. The common occurrence of PROM in Mulago Hospital labour ward implies that the adverse outcomes among women with PROM may also be common.

HSV-2 infections have been associated with preterm delivery [12, 13]. HSV-2 positive pregnant women had a twofold risk of preterm delivery compared to women who were HSV-2 negative [2]. Indeed, genital herpes DNA was demonstrated in both amniotic fluid and placental tissue of preterm deliveries [12, 14]. Similarly, women who recently seroconverted for HSV-2 had an increased risk of preterm delivery and babies with low birth weight [15].

Women who shed HSV-2 at delivery were more likely to have poor obstetric outcomes [16, 17]. Between 30 to 50% of HSV-2 positive women shed HSV in their genital tract during delivery period [16, 18]. In Soweto, South Africa, 17% of HSV-2 positive women shed HSV-2 at the time of delivery [19]. Herpetic Shedding is indicative of recent infection or reactivation [20] and has been associated with poor obstetric outcomes [2, 13]. Women who shed HSV-2 are more likely to transmit HSV-2 to their offsprings [20].

Given after 36 weeks, oral acyclovir suppresses HSV-2 genital shedding [21]. Acyclovir, a nucleoside analogue selectively inhibits herpetic DNA replication, leaving the uninfected cells unaffected [16, 21, 22]. Randomised placebo controlled trials among HSV-2 positive Women as diagnosed by culture and PCR demonstrated 90% reduction in genital herpetic shedding Moreover, acyclovir was found safe for use during pregnancy [21, 22].

The previous studies used acyclovir after 36 weeks until delivery to reduce genital shedding among populations with low HSV-2 prevalence. However, it was not clear to what extent acyclovir use from 28 to 36 weeks would reduce on the risk of PROM and other adverse obstetric outcomes in a setting with high HSV-2 prevalence. Uganda has a sero-prevalence of HSV-2 of 49.0% among women of reproductive age and over 60.0% among pregnant women [23]. Because ACV reduces HSV-2 in the genital secretions, we postulated that administration of acyclovir would reduce inflammatory mediators and undesirable outcomes such as PROM and preterm delivery. This study also aimed to validate the previous studies that showed a significant effect of acyclovir given from 36 weeks but never documented whether there was an added effect if the drug was given earlier in pregnancy. The primary objective of this study was to determine the effect of oral acyclovir administered from 28 to 36 weeks of pregnancy on the risk of preterm premature rupture of membranes. We also determined the effect of acyclovir administered from 28 to 36 weeks of gestation on the risk of other adverse obstetric outcomes comes (maternal puerperal sepsis, HSV-2 genital shedding at term, fetal demise, preterm delivery, low birth weight, low Apgar score and admission to neonatal intensive care unit) in Mulago Hospital.

## Methods

### Trial design

The study was a double blind (balanced randomized, one: one) placebo controlled trial among HSV-2 positive pregnant mothers attending antenatal clinic in Mulago Hospital, Uganda from January 2014 to February 2015.

### Study setting

The trial was conducted in the antenatal clinic for high-risk women, antenatal ward and the labour ward in the Mulago Hospital department of Obstetrics and Gynaecology, Uganda. Mulago Hospital serves as the Makerere University Medical School teaching hospital as well as a Ugandan National Referral Hospital. The hospital sees about 4000 women antenatally and delivers about 2000 women per month. More than 90% of pregnant women in Uganda book antenatal yet only 50% of them attend all the four required visits. The median gestational age of booking for antenatal care is 20 weeks of gestation.

Management of PROM at any gestational age in Mulago Hospital starts with making a diagnosis. The women usually complain of drainage or gush of fluid from the vagina before onset of labour. The diagnosis of PROM is made at speculum examination if there is observation of fluid pooling in posterior vaginal fornix or free flow of fluid from the cervix. All women with PROM are admitted. Antibiotics are given (Erythromycin 500 mg 6 hourly orally or Ampicillin 1 g 12 hourly intravenously or a cephalosporin intravenously ± if foul discharge- metronidazole 500 mg 8 hourly intravenously). The mother and fetus are assessed for infection: temperature, pulse, tenderness over uterus, altered color of liquor, full blood count, and obtain samples for cultures if indicated (fever, leukocytosis, foul smell or altered color discharge). Digital vaginal examinations are avoided until induction is indicated or as part of monitoring during active phase of labour. If the fetus is dead at any gestational age and there is no contraindications to vaginal delivery, induction of labour is performed immediately. If there is a contraindication to vaginal delivery (two or more P/S or ant uterine surgery, mal-presentation, mal-position, placenta praevia), then a caesarean-section is performed. If the baby is alive for a mother with PROM at term with no contraindication to vaginal delivery, induction of labour is performed. If the woman has contraindication to vaginal delivery then a caesarean section is performed.

Women with PROM at gestations above 28 weeks up to 36 weeks with a live foetus are given steroids to promote maturation of the fetal lungs (Intra muscular dexamethasone 12 mg 12 hourly for 24 h). Delivery is then indicated for the women with PROM at gestations above 34 weeks up to 36 weeks.

For the women with PROM between 28 and 34 weeks, expectant management coupled with monitoring maternal and fetal wellbeing on the ward is then done until 34 weeks provided there is no suspicion of intrauterine infection. An ultrasound is done for assessment of fetal position, cervical status and fluid volume once weekly. Antibiotics are continued for 5 days. Surveillance for infection continues and if infection occurs, intravenous antibiotics are given and delivery is indicated. Induction or caesarean section is done as per assessment by obstetrician on duty.

After delivery, antibiotics are given for 24 h in patients who have no evidence of infection. In the women who have evidence of infection, antibiotics are continued for 5 days. The babies are monitored for infection and are taken to the neonatal care unit if there is evidence of infection.

### Study participants

#### Screening for HSV-2 serostatus

We screened consented women between 20 and 26 weeks of gestation for HSV-2 serostatus as determined by HerpeSelect HSV-2 ELISA (Focus Diagnostics, CA, USA). Two milliliters (ml) of blood was drawn from each study participant into plain vacutainers (BD, Biosciences) and transported to the testing laboratory i.e. Immunology Laboratory at the Makerere University College of Health Sciences. At the laboratory, one ml of serum was recovered same day, and stored at 2–8 °C when the ELISA tests were to be done within 48 h or at −80 °C for samples that were to be tested thereafter At the time of performing the test, the stored serum samples were placed on the work table for about 1 h to bring them to room temperature. One tenth of the sera was mixed with 100 microliters of sample diluent prior to being tested for HSV-2 IgG following the manufacturer’s instructions [24]. The HerpeSelect HSV-2 ELISA has a sensitivity of 96–97% and a specificity of 98% as per the manufacturer [24]. We considered the cut off optical density of 3.5 and above as positive and below that as negative as has been the practice in recent studies in our setting following sensitivity and specificity studies [25].

#### Inclusion and exclusion criteria into the trial

We included consented HSV-2 sero-positive pregnant women aged 18–43 years at 28 weeks of gestation, at any parity and attending Mulago Hospital antenatal care clinic. Twenty-eight weeks is the lowest age of fetal viability according to Uganda Ministry of Health guidelines (Essential Maternal and Neonatal Care Clinical Care Guidelines for Uganda, July 2009). At least one in two babies born at or after 28 weeks survive without long time complications in Uganda [26]. We excluded one woman who had active genital herpetic lesions (needed higher dose of ACV) and one other woman who had high medication burden (had both HIV and Diabetic treatment). We also did not include those who returned after 28 weeks.

### Interventions

Enrolled participants were randomly assigned in a one to one ratio to take 400 mg of oral acyclovir twice a day or matching placebo from 28 weeks until 36 completed weeks using random computer generated numbers. The participants were told to take orally the tablets daily and to bring remaining tablets back at the next visit.

#### Similarity of Intervention

The acyclovir was purchased from Laborex pharmacy that was licensed to sell drugs in Uganda and was on the procurement list of Makerere University. Acyclovir was already approved by the National drug authority (NDA) and could be used if indicated among pregnant women after the first trimester. The placebo was a plain tablet containing sodium starch glycolate, magnesium stearate and maize starch with white coating formulated to have an external appearance similar to the acyclovir tablets. Both drugs were packaged by the Kampala Pharmaceuticals Industry (KPI) Limited into similar containers indistinguishable except by random numbers’ label.

### Randomisation

A pharmacist at KPI allocated the acyclovir or placebo using the random computer generated list. The randomisation sequence was produced using an online research randomiser in the 1:1 ratio by a statistician who did not take part in the study. The allocation was such that, each participant was enrolled and received a study number from research assistants. The participants were then escorted to the designated study pharmacy section where a corresponding envelope was opened. That envelope had a random code that corresponded with the code on a corresponding drug container which was then given to the client to take daily.

### Sample size calculation

The sample size for this study was determined for the primary outcome of preterm premature rupture of membranes (Pre-PROM) using Open Epi online sample size calculator. The formula was underlied by the Fleiss formula for determining sample size in comparative studies in which the outcome is a proportion (Fleiss et al. 1980), (Statistical Methods for Rates and Proportions, formulas 3.18 &3.19 [27]. The proportion of PROM without intervention was 12% which was the proportion of patients with PROM daily in the labour ward as per departmental records 2012 [10]. The expected proportion of the outcome in the intervention group was taken as 1.2% (acyclovir use reduced genital shedding from 34-37% to 2-4% which was over 90% reduction) [16, 28]. The study used 95% confidence interval and power of 80%. The sample size calculated was 83 in each of the arms. We added an anticipated 20% (17 participants to each arm) as loss to follow up thus giving us 100 women in the acyclovir and 100 in the placebo arms.

### Sampling procedure

To obtain the required sample, a consecutive sampling approach for pregnant mothers who met the inclusion criteria was done until the required sample was realized.

### Blinding

The researcher, research assistants and the participants were blinded to what the participant was receiving (acyclovir or placebo). It was only the pharmacist (who did the packaging) and the statistician (with the randomisation sheet) who knew what was packed.

### Outcomes

The primary outcome was development of premature rupture of membranes before or at thirty-six completed weeks. The secondary outcomes were preterm delivery, maternal, puerperal sepsis, HSV-2 genital shedding at term, fetal demise, low birth weight, low Apgar score (below 7 at 5 min), admission into neonatal special care unit and drug side effects. PROM was diagnosed from history of passage of clear fluid per vagina and confirmed by sterile speculum examination [6].

Preterm delivery is the birth of a baby before 37 completed weeks of gestation [6]. Maternal puerperal sepsis is infection of the genital organs and birth canal from the time of rupture of membranes or onset of labour up to the end 6 weeks after delivery [29]. The clinical features of puerperal sepsis include foul per vaginal discharge, abdominal pain and fever with temperature of 38.5 °C [6]. In our study we took puerperal sepsis as history of abnormal per vaginal discharge and fever which we noted after 24 and 72 h of delivery. Low birth weight was taken as weight of babies below 2500 grams.

HSV-2 genital shedding at term was taken to be evidence of HSV-2 DNA from cervical swab using PCR taken at 38 weeks whether in labour or not or earlier in case of preterm delivery. The PCR procedure was similar as was done in previous studies [30]. Specifically, DNA was extracted from the cotton swabs using phenol chloroform/Iso-amyl alcohol [31]. HSV-2 was then identified using primers and probes that use FluoroType HSV-2 software, version 1.0 (Hain LifeScience GmbH, Nehren, Germany). In this method, 6 microlitres of extracted DNA was added to 10 microliters of amplifier mixes (A and B) and a single stranded probe that emitted colour when HSV DNA was in the vicinity (Hain LifeScience GmbH, Nehren, Germany). The coloured probe was then heated by a Fluorocycler® and the intensity of emitted colour was then measured while the temperature rose [32]. The herpetic nucleotide sequences have different melting points which are plotted as curves [32]. The sequence detector software interpreted the presence or absence of peaks of the melting points corresponding to HSV-2 DNA giving positive or negative results respectively (Hain LifeScience GmbH, Nehren, Germany).

The drug side effects assessed included: visual blurring, dizziness, drowsiness, nausea, vomiting, sensitivity to sunlight, hypersensitivity rash, fever, hallucinations and blood in urine. The other effects assessed were abnormal levels of hemoglobin, liver alanine transferase and creatinine by the time of preterm rupture of membranes or 38 weeks.

The independent variables included socio-demographic characteristics (age, marital status, highest level of education attained, occupation and parity); obstetric history such as past history of premature rupture of membranes, HIV status obtained from antenatal hospital records and concurrent candidiasis and *Trichomonas vaginalis*.

### Study procedures and Follow up

Baseline socio demographic and obstetric data was collected and recorded onto interviewer administered case report forms for the enrolled participants by midwives who were trained in the research procedures. HIV serostatus was obtained from the antenatal records. An obstetric examination was done followed by a pelvic examination and then two vaginal swabs were taken off for candidiasis and *Trichomonas vaginalis*. Four millilitres of blood was drawn for renal function, liver functions and full haemogram.

Study visits were conducted at 32, 36, 38 weeks and delivery. The 32-week visit was 4 weeks after enrollment and served as routine antenatal care as per the guidelines for the Ministry of Health and for refill of study drug with similar random code. At the 36-week visit the following were done: antenatal care, review on completion of the randomised intervention study period and giving of routine suppressive acyclovir. During the visits at 32 and 36 weeks, compliance with drugs and complaints were assessed. Compliance was measured by self-report of the duration the study medication was taken. In addition, any concomitant medications were noted and documented. Furthermore, relevant investigations were done. All patients were given iron and folic acid for oral intake. In addition, Sulphadoxine/pyrimethamine (Fansidar^TM^) for prevention of malaria was given as per the Uganda Ministry of Health guidelines (Essential Maternal and Neonatal Care Clinical Care Guidelines for Uganda, July 2009). For all participants, relevant documentation was done at every visit.

The women who developed PROM were given antibiotics and managed according to the protocol of the hospital adapted from that of Society of obstetricians and gynaecologist for Canada [6]. Complaints of fever and abnormal vaginal discharge were assessed at 24 and 72 h after delivery. In addition, fetal demise, low birth weight, Apgar score and admission into neonatal special care unit were also recorded onto the relevant forms. We took cervical swabs for HSV-2 DNA PCR at 38 weeks/or before that if the participant got preterm delivery or rupture of membranes. For the women who missed the 38-week visit we did the cervical swab at any time they came in labour. The full haemogram, renal and liver function tests were repeated at closing visit. Any complaints were recorded on an adverse event form. Serious adverse events (SAEs) were predetermined as: admission of baby in neonatal unit for more than 24 h, fetal death, plus readmission of the mother during puerperium or maternal death. The SAEs were to be recorded onto a serious adverse form but we got none. Any drugs that were prescribed and dispensed to the participants were recorded onto the concomitant drug log.

### Data management, quality control and statistical analysis

All the research assistants had training in ethical conduct of research, the consent process and data collection a week preceding to the study. There were also weekly study meetings to review progress of enrollment and challenges met. Relevant refresher training was done as the need arose. The questionnaires were piloted. Accredited laboratories were used for study investigations. The data was checked daily for exhaustiveness by the doctor on research duty and the Principal Investigator. Queries were raised daily and resolved. Double data entry was done using the EPI- INFO programme. Data was then exported to STATA version 12 (Stata Corp., College Station, TX, USA) for analysis.

An independent Data and Safety Monitoring Board (DSMB) was established to oversee the safety and progress of the trial. If a serious adverse event (maternal or fetal readmission or a death) occurred, it was to be reported within 24 h to the DSMB then to the HDREC and UNCST. None of the participants reported a serious adverse event thus, no SAE reports were made.

#### Interim analysis and review by the DSMB

Interim analysis was done when we had accumulated data for 30% of sample size (60 participants). There were no reports of serious adverse events in either arm. There were no differences in the acyclovir arm compared to the control arm as regards adverse outcomes so the study was allowed continuation to completion.

Intention to treat analysis was used. All randomised participants were included in the analysis and were retained in the group to which they were allocated. The primary endpoint was development of rupture of membranes by 36 weeks.

#### Descriptive statistics

Categorical data for intervention and placebo groups were summarized as counts and proportions while continuous variables such as age were summarized using mean and standard deviations.

#### Bivariate analysis

Comparison between the intervention groups and placebo group on the basis of baseline characteristics was done. Computation and comparison of the incidence of PROM by 36 completed weeks in the acyclovir, placebo arm were done by using risk ratios, and the significance of the risk ratios was assessed by the *p*-values and 95% confidence intervals.

Computation and comparison of the percentage of patients who developed secondary outcomes in the intervention group and placebo group was done by using risk ratios and the significance of the risk ratios was assessed by the *p*-values and 95% confidence intervals.

### Ethical consideration

Ethical approval for the study protocol, case report forms and consent forms was obtained from the Makerere University College of Health Sciences School of Medicine Research and Ethics Committee and the Uganda National Council of Science and Technology (approval number HS 1413). We acquired written informed consent from all the participants who were screened and enrolled. All participants had health education about HSV-2 prevention. All participants who reached 36 weeks of gestation were given acyclovir to prevent their babies from acquiring HSV-2 perinatally. The data collected contained sensitive information on sexual practices, thus confidentiality was observed for all the study documentation. The records were placed under double lock and key. The data bore participant initials and not names.

## Results

**Fig. 1:**
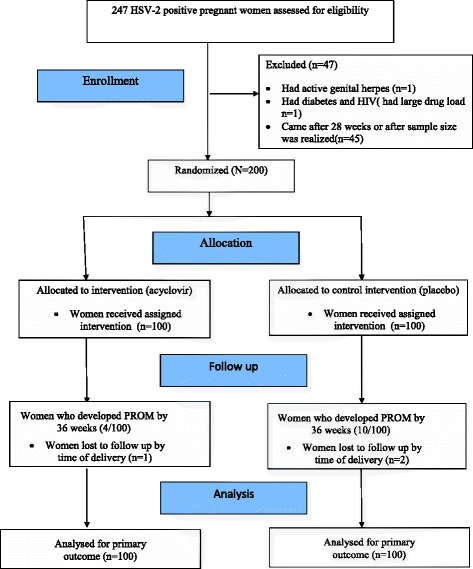
Trial flow diagram showing the participants flow and numbers from enrollment, allocation, follow up and analysis between January 2014 and February 2015. Two hundred and forty seven HSV-2 positive women were eligible for enrollment. Forty-seven women were excluded. One participant had active genital herpes and needed a therapeutic course of acyclovir. One woman had diabetes and HIV and had large drug load. Forty-five participants came after 28 weeks or after sample size was realised. We thus had the 200 participants who were eligible and randomised half of them in the acyclovir arm and the other half in the placebo arm. We followed up all participants to 36 weeks or up to membrane rupture, if it occurred before that and the primary outcome was assessed then

We screened 531 pregnant women for HSV-2; of these 247 were HSV-2 positive and thus eligible for enrollment. We enrolled 200 eligible women of which 100 were assigned randomly to the acyclovir arm as well as 100 to the placebo arm. We assessed primary outcome by 36 weeks. We continued routine visits for all women who were undelivered by 36 weeks up to delivery. The details of the enrollment and the randomisation are in Fig. 1 as per the Consolidated Standards of Reporting trials (CONSORT) guidelines.

### Baseline characteristics

The intervention and the placebo arms were comparable by age, highest education achieved, marital status, gravidity, HIV status, past history of premature rupture of membranes, occupation of participant, occupation of partner and highest education of partner (Table 1).

**Table 1:** Shows that the distribution of age, gravidity, education level, marital status, occupation and religion of 87 cases and 87 controls was similar between January 2014 and February 2015

Characteristics	Acyclovir arm *n* = 100 (%)	Placebo arm *n* = 100 (%)	*P* value
Mean age in years (SD)^a^	28.5 (5.4)	28.3 (5.5)	0.727
Education			
Less or equal to Primary	27 (27.0)	39 (39.0)	0.07
Secondary and above	73 (73.0)	61 (61.0)	
Marital status			
Not married	6 (6.0)	7 (7.0)	0.774
Married/cohabiting	94 (94.0)	93 (93.0)	
Occupation			
Business	26 (26.0)	28 (28.0)	
Employed/professional	42 (42.0)	38 (38.0)	0.356
Unemployed	32 (32.0)	34 (34.0)	
Occupation of partner			
Business	38 (38.0)	37 (37.0)	
Employed/professional	62 (62.0)	61 (61.0)	0.356
Unemployed	0(0.0)	1 (1.0)	
Education of partner			
Less or equal to Primary	21 (21.0)	28 (28.0)	0.250
Secondary and above	79 (79.0)	72 (72.0)	
Gravidity			
Prime	5 (5.0%)	4 (4.0)	
Parous	80 (80.0)	74 (74.4)	0.609
Multipara	15 (15.0)	22(22.0)	
HIV status			
Negative	83 (83.0)	76 (76.0)	0.220
Positive	17 (17.0)	24 (24.0)	
Past premature rupture of membranes			
None	93 (93.0)	93 (93.0)	1.000
Yes	7 (7.0)	7 (7.0)	

There were no significant differences in the distribution of socio demographics characteristics of 200 participants and of their partners in the intervention and placebo arms. There were also no significant differences in the distribution of gravidity, HIV serostatus and past history of ruptures of membranes of participants in the intervention and placebo arms

^a^Here means and standard deviation were used

### Numbers analysed

All the women had the primary outcome analysed by 36 weeks. The compliance to the study medications was similar in both arms. About 90% of the study participants in each arm took the study medication for at least 4 weeks. However, only 50 and 54% of the participants in the acyclovir arm and in the placebo arm respectively complied fully with the study medication schedule. The women who complied with the medication were similar in baseline characteristics compared to the women who did not comply with the study medication; 28% (30/109) and 34% (34/91) of the compliers compared to the non-compliers respectively had primary education, *p* = 0.844. Six percent (6/109) and 8% (7/91) of the compliers compared to the non-compliers respectively were not married, *p* = 0.553. Twenty eight percent (31/109) and 25% (23/91) of the women who complied compared to those who did not comply to the study medication respectively were business women, *p* = 0.153. Fifteen percent (16/109) and 27% (25/91) of the women who complied compared to those who did not comply to the study medication respectively were HIV positive, *p* = 0.153.

### Outcomes

The obstetric outcomes per arm are shown in Table 2. There was no statistically significant difference in the acyclovir arm and placebo arm by incidence of preterm premature rupture of membranes by 36 weeks (4.0% versus 10.0%; RR 0.35; 95% 0.11–1.10), HSV-2 shedding (10.3% versus 12.0%; RR 0.55; 95% 0.22–1.42) and low birth weight (8.0% versus 15.0%; RR 0.43; 95% 0.18–1.02). There was a significant reduction in the acyclovir arm compared to placebo arms by incidence of preterm delivery (11.1% versus 23.5%; RR 0.41; 95% 0.20–0.85) and admission to special care unit (9.0% versus 17.3%; RR 0.43; 95% 0.19–0.96). There was no record of symptoms suggestive of puerperal sepsis among the participants in either arm. There was also no record of fetal death by 72 h or Apgar scores less than 7 by 5 min among babies of participants in either arm.

**Table 2:** Obstetric outcomes per study arm for HSV-2 positive pregnant participants in Mulago Hospital between January 2014 and February 2015

Obstetric out comes	Acyclovir *N* =100(%)	Placebo *N* =100(%)	Relative Risk (RR) (95%CI)	*P* values
Total				
PROM by 36 weeks				
No	96 (96.0)	90 (90.0)		
Occurred	4 (4.0)	10 (10.0)	0.35 (0.11–1.10)	0.073
Preterm delivery (<37 weeks)^a^				
No	88 (88.9)	75 (76.5)		
Occurred	11 (11.1)	23 (23.5)	0.41 (0.20–0.85)	0.016
Low birth weight^a^				
No	91 (92.0)	83 (85.0)		
Yes	8 (8.0)	15 (15.0)	0.43 (0.18–1.02)	0.056
Admission in special care unit^a^				
No	90 (91.0)	81 (82.7)		
Yes	9 (9.0)	17 (17.3)	0.43 (0.19–0.96)	0.040
Evidence of shedding at term^b^				
No	70 (89.7)	73 (88.0)		
Occurred	8 (10.3)	10 (12.0)	0.55 (0.22–1.42)	0.215

All the 200 participants had PROM by 36 weeks assessed. Ninety-nine and ninety-eight participants in the acyclovir arm and placebo arms respectively had the delivery outcomes assessed. We were unable to trace the outcomes for the 3 remaining participants. Thirty-nine participants missed the 38 week visit (17 in the placebo and 22 in the acyclovir arm) and delivered elsewhere. As stated above, we were able to get delivery outcomes for all except three. Unfortunately, we were unable to have cervical swabs taken at delivery for those 39 participants and thus had no results for genital shedding for them. Acyclovir between 28 and 36 weeks did not reduce the incidence of preterm PROM by 36 weeks HSV-2 shedding by delivery time and low birth. However, acyclovir administered between 28 and 36 weeks reduced the incidence of preterm delivery and admission to special care unit

^a^Here the numbers do not add up to 100; Acyclovir arm has 99 and placebo arm 98

^b^Here the numbers do not add up to 100; Acyclovir arm has 78 and placebo arm 83

### Drug reactions

There were no differences in adverse drug reactions between the two arms. There were no reports of visual blurring, sensitivity to sunlight, un-explained fever, hallucinations or of blood in urine in either arm. There were no serious adverse effects. Dizziness, drowsiness, and skin rash were negligible in either arm. Side effects such as nausea, vomiting, abnormal blood counts, and liver and renal function tests were similar by study arm (Table 3). Patients with haemoglobin less than 11.9 mg per decilitre were similar in both arms (21.7 and 22.9% for acyclovir and placebo arms respectively, *p* = 0.846). Patients with abnormal creatinine levels were similar in both arms (1.1 and 5.3% for acyclovir and placebo arms respectively, *p* = 0.105). Patients with abnormal liver alanine transferase were also similar in both arms (3.3 and 1.1% for acyclovir and placebo arms respectively, *p* = 0.297).

**Table 3:** Side effects per study arm for HSV-2 positive pregnant participants in Mulago Hospital between January 2014 and February 2015

Characteristic	Acyclovir *N* =100(%)	Placebo *N* =100(%)	*P* values
Total			
Nausea and vomiting			
None	93 (93.0)	96 (96.0)	
Yes	7 (7.0)	4 (4.0)	0.352
Hb level^a^			
Normal	72 (78.3)	74(77.1)	
Abnormal Less than 11.9 g/dl	20 (21.7)	21 (22.9)	0.846
Creatinine level^a^			
Normal	91 (98.9)	90 (94.7)	
abnormal	1 (1.1)	5 (5.3)	0.105
Alanine transferase (ALT)^a^			
Normal	89 (96.7)	94 (98.9)	
abnormal	3 (3.3)	1 (1.1)	0.297

We were able to assess history of nausea and vomiting for all the study participants. Thirteen participants declined the second blood draw, thus we only assessed blood counts, renal functions tests and liver function tests for 92 and 95 participants in the acyclovir and placebo arms respectively. There were no differences in adverse drug reactions between the acyclovir and placebo arms

^a^Here the numbers are not adding up to 100 due to some participants declining closing laboratory tests blood draws; 92 in the acyclovir arm and 95 in the placebo arm

## Discussion

This was a randomised double blind placebo controlled trial comparing use of oral suppressive acyclovir and placebo among HSV-2 positive pregnant women from 28 to 36 weeks followed by standard care from 36 weeks up to delivery. The standard care among HSV-2 positive pregnant women includes use of oral acyclovir from 36 weeks up to delivery. We assessed whether there was reduction of incidence of preterm premature rupture of membranes (pre-PROM) primarily. We also assessed for reduction of maternal puerperal sepsis, HSV-2 genital shedding at term, fetal demise, preterm delivery, low Apgar score, low birth weight and drug side effects. Suppressive acyclovir from 28 to 36 weeks had no effect on the incidence of preterm PROM, puerperal sepsis, HSV-2 genital shedding, fetal demise, birth weight, Apgar score and drug side effects but significantly reduced preterm delivery and neonatal admissions into the new-born special care unit.

Oral suppressive acyclovir did not significantly reduce the incidence of preterm premature rupture of membranes by 36 weeks. This is in contrast to what is expected given that inflammation of the cervix occurs among patients with herpes infection with release of cytokines that are implicated in membrane rupture [3, 33]. We had theorized that that because acyclovir reduces genital shedding (both HSV-2 symptomatic reactivation and asymptomatic shedding) then it would also reduce incidence of premature rupture of membranes [16]. Our study showed no difference in HSV-2 shedding due to acyclovir administration from 28 to36 weeks compared to placebo administered for the same duration. However, the incidence of HSV-2 shedding in both arms was less than that seen in other studies at term without acyclovir (17% in Soweto (South Africa), 22% in Uganda and 34% in Washington and Vancouver) [16, 19, 34]. Our results further imply that, acyclovir given from 28 weeks up to delivery reduced HSV-2 shedding to a similar level as that when given from the standard 36 weeks onwards. We monitored compliance to drug therapy using self-report. Given that, full compliance to the study drug by the participants was only by 50% of the participants in each study arm, more studies with better compliance follow up may need to be done to explore the relationship between HSV-2 and PROM further.

Suppressive acyclovir significantly reduced incidence of preterm delivery (from 23.5 to 11.1%) and neonatal admissions into the special care unit (from 17.3 to 9.0%). Studies done over the years have associated HSV-2 positivity with preterm labour due to genital shedding of HSV-2 [13]. Acyclovir is known to reduce genital shedding, thus we had postulated that acyclovir administered from the week 28 to 36 weeks would reduce the incidence of preterm delivery [16, 21]. Given that, the reduction of HSV-2 shedding was similar in the intervention and placebo arms, our results imply that the effect of acyclovir reduction on incidence of preterm delivery may also have another mechanism independent of herpetic shedding. In fact, herpetic viruses have been associated with placental pathology leading to pregnancy induced hypertension in epidemiological studies [2]. Pregnancy induced hypertension is a common cause of preterm births [6]. The effect of acyclovir on low birth weight was marginal (from 15.0 to 8.0%) and was probably also related to the preterm delivery. There is thus a need to look at markers of preterm delivery among HSV-2 positive women in order to document reduction of the levels of these markers when acyclovir is used for different durations.

From our study, some of the adverse outcomes associated with rupture of membranes such as puerperal sepsis did not occur probably due to vigilance by the study team to follow the protocol for use of antibiotics with patients with PROM [6]. In addition, both study arms did not report fetal death by 72 h and Apgar sores less than 7 by 5 min because of the many patients delivering with skilled attendance. In Uganda 58% of patients deliver under skilled attendance but in our study close to 80% of the patients delivered in hospital [35]. Furthermore, care in public facilities has greatly improved in Uganda which also may have contributed to good Apgar scores [36].

### Limitations

We did not have evidence of HSV-2 PCR at enrollment into the study before starting suppressive antiviral therapy however; we used a cut off of 3.5 optical densities which has high specificity for HSV-2 antibody detection even for African populations. Our rationale is that during scaling up use of acyclovir suppressive therapy in low to middle income countries, the national ministries of health would find it cheaper to use HSV-2 serological tests rather that HSV-2 DNA PCR. The study was conducted in a national referral hospital setting which may not be generalizable to lower level health care settings. However, we generated useful information on the effect of acyclovir from 28 weeks compared to routine suppressive acyclovir at or after 36 weeks.

Full compliance to study drug was less than optimal but is comparable to the current national compliance to antenatal package by pregnant women. The results would thus still apply in the prevailing situations unless better compliance methods are explored and utilised. Good enough, there was no bias due to the less than optical compliance level to the study medication, given that baseline characteristics were comparable among the women who took the medication as prescribed compared to those who missed some medications. Using the cut off above 3.5 may have brought prevention to a population with higher risk of disease and could have skewed the results. However, the cut off of 3.5 gives sensitivity and specificity of over 95% which is comparable to western blot which is a Gold standard non-commercial test. We did not take into account the frequency of genital conditions such as bacterial vaginosis (BV); however, there was no association between BV and HSV-2 in one study by the same authors [10]. BV testing may have affected the results, we will thus consider interaction of other genital infections in other studies in this area of HSV-2 and obstetric outcomes.

We excluded women with active HSV-2 and those with high medication burden. Although it is true that women with active HSV-2 and those taking many medications may have benefitted from the study, someone with active HSV-2 would need a higher dose for acyclovir than that that used prophylaxis. The woman with active HSV-2 was thus excluded and given the appropriate acyclovir dosage. For ethical reasons when an eligible participant clearly indicated that she had too many drugs to take (ARVs and Insulin) and declined more drugs, we had to honour her desire. However, only two women were excluded for either having active HSV-2 or due to high medication burden. Exclusion of those two women likely did not affect the outcome of interest.

## Conclusions

Oral suppressive acyclovir given to HSV-2 positive pregnant women from 28 to 36 weeks did not reduce incidence of preterm premature rupture of membranes but reduced incidence of preterm delivery.

### Recommendations

We recommend use of suppressive acyclovir from 28 weeks by obstetricians on case to case basis in pregnant women with genital herpes to prevent premature delivery. However, we do not recommend the above suppressive regimen from 28 weeks to prevent preterm premature rupture of membranes solely.

## Abbreviations

ACV: Acyclovir
BV: Bacterial vaginosis
HIV: Human immunodeficiency virus
HSV-2: *Herpes simplex* virus type-2
PCR: Polymerase chain reaction
PROM: Premature rupture of membranes
Pre- PROM: Preterm premature rupture of membranes
